# Under age five children survival times in Nigeria: a Bayesian spatial modeling approach

**DOI:** 10.1186/s12889-022-14660-1

**Published:** 2022-11-28

**Authors:** Osafu Augustine Egbon, Mariella Ananias Bogoni, Bayowa Teniola Babalola, Francisco Louzada

**Affiliations:** 1 Institute of Mathematical and Computer Sciences, University of São Paulo, São Carlos, Brazil; 2 Department of Statistics, Universidade Federal de São Carlos, São Carlos, Brazil; 3 Department of Mathematics and Statistics, Kampala International University, Kampala, Uganda

**Keywords:** Hazard model, Bayesian hierarchical model, Under-five mortality, Survival probability

## Abstract

**Background:**

Nigeria is among the top five countries in the world with the highest under-five mortality rates. In addition to the general leading causes of under-five mortality, studies have shown that disparity in sociocultural values and practices across ethnic groups in Nigeria influence child survival, thus there is a need for scientific validation. This study quantified the survival probabilities and the impact of socioeconomic and demographic factors, proximate and biological determinants, and environmental factors on the risk of under-five mortality in Nigeria.

**Methods:**

The Kaplan-Meier survival curve, Nelson Aalen hazard curve, and components survival probabilities were estimated. The Exponential, Gamma, Log-normal, Weibull, and Cox hazard models in a Bayesian mixed effect hierarchical hazard modeling framework with spatial components were considered, and the Deviance and Watanabe Akaike information criteria were used to select the best model for inference. A $5\%$ level of significance was assumed throughout this work. The 2018 Nigeria Demographic and Health Survey dataset was used, and the outcome variable was the time between birth and death or birth and the date of interview for children who were alive on the day of the interview.

**Results:**

Findings show that the probability of a child dying within the first two months is 0.04, and the probability of a boy child dying before attaining age five is 0.106, while a girl child is 0.094 probability. Gender, maternal education, household wealth status, source of water and toilet facility, residence, mass media, frequency of antenatal and postnatal visits, marital status, place of delivery, multiple births, who decide healthcare use, use of bednet are significant risk factors of child mortality in Nigeria. The mortality risk is high among the maternal age group below 24 and above 44years, and birth weight below 2.5Kg and above 4.5Kg. The under-five mortality risk is severe in Kebbi, Kaduna, Jigawa, Adamawa, Gombe, Kano, Kogi, Nasarawa, Plateau, and Sokoto states in Nigeria.

**Conclusion:**

This study accentuates the need for special attention for the first two months after childbirth as it is the age group with the highest expected mortality. A practicable way to minimize death in the early life of children is to improve maternal healthcare service, promote maternal education, encourage delivery in healthcare facilities, positive parental attitude to support multiple births, poverty alleviation programs for the less privileged, and a prioritized intervention to Northern Nigeria.

## Background

The under-five mortality rate is still a persistent problem in the world. According to [1], the global number of under-five deaths was estimated as $5.2\, (5.0, 5.6)$ million in 2019, which implies that approximately 14, 000 children died every day before attaining their fifth birthday in 2019. Since 1990, the global under-five mortality rate has declined by $59\%$, dropping from 93 deaths per 1000 live births in 1990 to 38 deaths per 1000 live births in 2019, [2]. Although there is a substantial global reduction in under-five mortality rates since the year 1990, disparities exist across countries. Sub-Saharan Africa region remains the highest-burden region of under-five mortality in the world, with one in thirteen children dying before reaching the fifth birthday thus, placing the region at top risk region of under-five deaths, [2]. According to WHO, half of the global under-five deaths occurred in five countries, including Nigeria.

Nigeria is one of the countries that presented a high under-five mortality rate of 117 per 1, 000 live births in 2019, [3]. The country is listed among the top five countries with the highest under-five mortality rate in 2019. A collective effort of the government, stakeholders, and non-governmental organizations in the fight against under-five mortality to improve the well-being of children has led to a consistent decline in the early child mortality rate in Nigeria, [3]. Despite several interventions to lower the under-five mortality index in Nigeria, the rate is still relatively high [4]. The slow declining pace of the rate may hinder the attainment of the Sustainable Development Goal (SDG) to end preventable under-five death in 2030. The SDG target is to reduce under-five mortality to at least 25 per 1,000 live birth by 2030, [2]. Given the statistics, there must be at least a 50% decline in early childhood mortality in Nigeria to attain the SDG by 2030, [5]. Therefore, the under-five mortality burden in Nigeria calls for rapid attention to scale down and below the global mortality average rate.

Leading causes of under-five death include preterm birth complications, birth trauma, pneumonia, congenital anomalies, diarrhea, and malaria, [2, 6], which are preventable with affordable interventions. However, under-five death in Nigeria has been attributed to additional factors including, disparities in sociocultural values and practices, [7]. Cultural beliefs either positively or negatively influence the health status of children, and consequently introduced heterogeneity in improving the survival and well-being of children. For instance, the large disparity of birth in health facilities, births attended by qualified medical practitioner, number of antenatal and postnatal care utilization across different ethnic groups can be attributed to the heterogeneity in the country, [8–10]. Nonetheless, there are enormous geographical variations in the under-five mortality rate in the country. According to [5] report, most under-five deaths in Nigeria occurred in the Northern states. Thus, there is a need for scientific validation of the contribution of socioeconomic and demographic factors increasing the risk of child mortality in Nigeria.

Several types of research have been conducted to contribute to the fight against child mortality in Nigeria. For instance, [11] extensively discussed specific medical solutions to reduce neonatal mortality in Nigeria. The authors suggested the adaptation of imported medical technologies through scientific thinking, as many imported technologies may underperform given the environmental impact. Jones et al. [12] investigated the impact of several interventions on child mortality and estimated the scope of coverage of these interventions. According to the authors, there are sufficient medical knowledge and instruments to reduce child mortality, but the mortality rate is still high because the injection of these interventions into the population has not fully considered the inequalities and heterogeneity among the children. Hence, policies and intervention programs should be aware of the factors responsible for these inequalities. In the attempt to determine these factors, [7] examined the effect of ethnicity on under-five mortality in children and found that the risk of mortality significantly varies between ethnic groups in Nigeria. It was found that the mortality rate is high among Hausa, Fulani, and Kanuri tribes in Nigeria. Angela and Uju [13] investigated the effect of child characteristics on under-five mortality in Nigeria. They found that birth between the maternal ages of 20-24 has a lower risk of mortality. Adeyinka et al. [4] determined the socioeconomic indicators contributing to child mortality in Nigeria, and surprisingly, concluded that attendance of skilled health workers during delivery was associated with an increased risk of neonatal death. Ayoade [14] examined the Spatio-temporal distribution of the under-five mortality rate in Nigeria and found that there is spatial clustering and geographical disparities between states in Nigeria. Recently, [15] examined the effect of spatial distribution and other covariates on under-five mortality deaths in Nigeria.

Many researchers adopted the indirect method of estimating mortality rates in Nigeria, where the survival status of children in a cohort is used to estimate the mortality rate. The implicit assumption of this approach is that the births of a cohort are the number of children born in a period, [16]. This assumption is hardly satisfied as studies have shown a disparity in a maternal age group to the risk of under-five mortality, [17]. Moreover, an approach commonly adopted in these researches in multilevel modeling of under-five mortality rate is to consider the death status of a child as a binary outcome variable, [15]. In this manner, information about the time localization of mortality risk is lost in the process. Thus, give rise to a poor time resolution of under-age five mortality and could lead to biased estimates of the variance, [18, 19].

This study aims to quantify the survival probabilities and the impact of socioeconomic and demographic factors, proximate and biological determinants, and environmental factors on the risk of under-five mortality in Nigeria. A direct estimation method of mortality rates was adopted, where the date of birth, survival status, and ages at death of children was utilized for the analysis. In this manner, the heterogeneity in the mortality risk between ages 0 to 59 months is incorporated for inference. For instance, a heap of under-five deaths a few months after birth can be incorporated into the modeling framework for estimating the risk in these age groups. Moreover, this study accounted for the uncertainty in the geographical heterogeneity of under-five mortality rates between the states in Nigeria. Nonparametric survival models and parametric hazard models within a Bayesian hierarchical modeling framework were used to quantify the survival probabilities and the impact of risk factors and spatial patterns on child survival before the fifth birthday. This would unravel the temporal survival pattern and give insight into the existing survival inequalities among children across the country, which could be valuable for policy-making and strategic

## Materials and method

### Study area and data collection

**Fig. 1:**
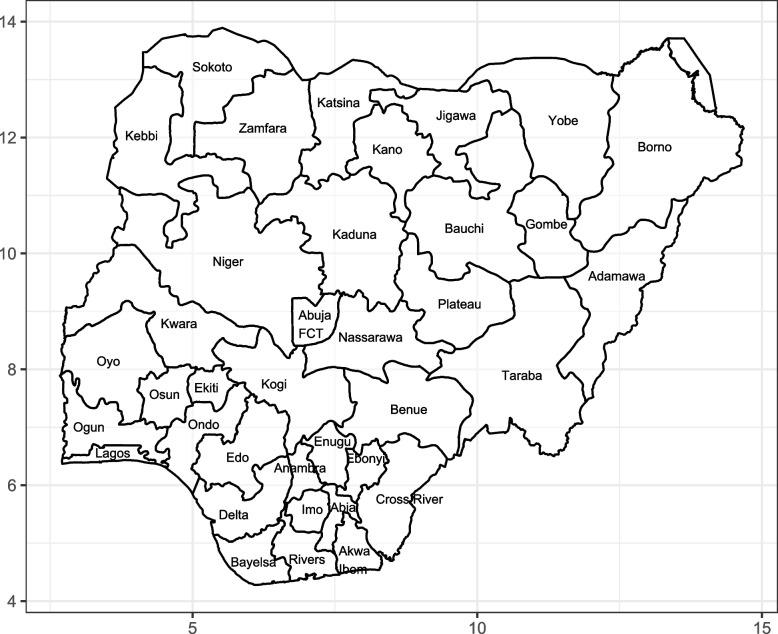
Map of Nigeria

Nigeria is a west African country with 36 administrative states and a Federal Capital Territory (FCT). Figure 1 shows the map of Nigeria, which served as the study area. The data used were pooled from the 2018 Nigeria Demographic and Health Survey (NDHS), implemented by the National Population Commission (NPC). The survey took place between 14th August and 29th December 2018. The sampling frame adopted is the population and Housing Census conducted by the NPC in 2006. The Enumeration Areas (EA) in the 2006 Census served as the survey’s primary sampling units. The survey implemented a two-stage stratification sampling technique to include EAs in the selection. In the first stage, samples of 1400 EAs were drawn with probability proportionate to size. The resulting listing of households served as the sampling frame in the second stage. A total of 40,427 women were interviewed successfully, yielding a response rate of 99%. Children born within the last 59 months to the interview day were eligible to be included in the study. The age at death of children and the present age of children alive on the interview day was of prime interest. This analysis included a record of 33, 697 children after data cleaning.

### Study variables

The outcome variable is the survival times of under-five children within a five years study frame, which is composed of the time between birth and death, $t_d$, and the time between birth and the date of interview, $t_a$, if the child was alive at the interview day. Mathematically, $t_d={\text {Age at death (in years)}}/{12}$ and $t_a={[\text {Date of interview}-\text {Date of birth (in years)}]}/{12}$. The independent variables included in the hazard model can be categorized into socioeconomic and demographic factors, proximate and biological factors, and environmental factors. These variables are presented in the Appendix. According to the World Health Organization classification, the environmental factors such as household’s toilet facility and source of water supply, were classified as either improved or unimproved. The improved toilet facility includes flush or pour-flush to a piped sewer system, septic tank or pit latrine, ventilated improved pit latrine, pit latrine with slab, and composting toilet. Similarly, the improved water source includes piped household water connection inside the users dwelling, public taps or standpipes, tube wells or boreholes, protected dug wells, protected springs and rainwater collection, [20].

### Data analysis

#### Nonparametric method

Let $n_i$ be the number of children included in state *i*, then, $n=\sum _{i=1}^{37}n_i=33,697$ is the number of children under study and with $k\le n$ distinct time of deaths between birth and time of the survey, such that $t_1 \le t_2 \le ...\le t_k$. Note that there is a possibility of having more than one death at each event time. Denote $d_i$ as the number of deaths at time $t_i$, and $q_i$ as the number of children at risk at time $t_i$. Thus the Kaplan-Meier estimator of the survival function *S*(*t*), and the Nelson-Aalen estimator of the cumulative Hazard function *H*(*t*) are given as

1 $$\begin{aligned} \hat{S(t)}=\prod\limits_{t_i:t_i\le t}^{}\Bigg (1-\frac{d_i}{q_i}\Bigg ),\,\, \hat{H(t)}=\sum\limits_{i:t_i< t}^{}\Bigg (\frac{d_i}{q_i}\Bigg ). \end{aligned}$$

The $100(1-\alpha )\%$ confidence interval of *S*(*t*) is given as $\hat{S(t)}\pm z(\alpha /2)\sqrt{V( \hat{S(t)})}$, where

2 $$\begin{aligned} V(\hat{S(t)})=\left( \hat{S(t)}\right) ^2\sum\limits_{i:t_i< t}^{}\frac{d_i}{q_i(q_i-d_i)}. \end{aligned}$$

For the description of the computational steps for DHS component survival probabilities see [16, 21].

#### Parametric method

Let $C_{ij}$ be a random variable representing the number of days between the birthday of child *j* in state *i* and the day of the interview. Let $t_{ij}$ be the number of days a child lived until the event of interest occurred. Define $y_{ij}=\min (t_{ij},C_{ij})$, and $\delta _{ij}\in \{0,1\}$ be a censoring indicator that takes the value 1 if $t_{ij}\le C_{ij}$ (i.e child *j* in state *i* experienced the event of interest (death) and takes the value 0 otherwise). Let *Y* be a random variable that takes the value $y_{ij}$ for $j=1,2,...,n_i$, and $i=1,...,37$ with density function $f(y_{ij}|\lambda )$. The survival probability is given as

3 $$\begin{aligned} S(y_{ij}|\varvec{\lambda }) =\int _{y_{ij}}^{\infty } f(x|\varvec{\lambda })d x, \end{aligned}$$

which is equivalent to $S(y_{ij}|\varvec{\lambda })=\frac{f(y_{ij}|\varvec{\lambda })}{h(y_{ij}|\varvec{\lambda })}$, where *h* is the hazard function of *Y*. Thus the likelihood function to incorporate the incomplete information is given as

4 $$\begin{aligned} L(Y|\varvec{\lambda }) = \prod\limits_{i=1}^{37}\prod\limits_{j=1}^{n_i} f(y_{ij}|\varvec{\lambda })^{\delta }S(y_{ij}|\varvec{\lambda })^{1-\delta }=\prod\limits_{i=1}^{37}\prod\limits_{j=1}^{n_i} f(y_{ij}|\varvec{\lambda })^{\delta }\Bigg (\int _{y_{ij}}^{\infty } f(x|\varvec{\lambda })d x\Bigg )^{1-\delta }. \end{aligned}$$

Let $\eta$ be a linear predictor, which is a function of demographic and socio-economic variables, and represent $\eta =g(\lambda )$, where *g* is a known link function usually determined by the density function of *Y*. The model is specified through the hazard function of *Y*. Consequently, the likelihood function is written as $L(Y|g^{-1}(\eta ))$. In the case of a two parameters model with location and scale parameters, $\lambda _1$, and $\lambda _2$ respectively, $\eta =g(\lambda _1)$ and the likelihood is given as $L(Y|g^{-1}(\eta ),\lambda _2)$.

In this study, four probability density functions were considered for *Y*, namely, the Exponential, Gamma, Weibull, and Log-normal probability density functions. In addition, the Cox proportional hazard model was also adopted. The summary of the five competing models and their properties for a single child is presented in Table 1.

**Table 1:** Competing parametric models. $\Lambda$ is the parameterized Gamma density, and $\gamma (.,.)$ is the lower incomplete gamma function

Model	Parameterized Density	Hazard Function	Link	Restriction
Exponential	$\lambda \exp (-\lambda y)$	$\lambda$	$\eta =\log (\lambda )$	$\lambda >0$
Gamma	$\frac{1}{\Gamma (s\phi )}\left( \frac{(s\phi )}{\mu }\right) ^{(s\phi )}y^{(s\phi )-1}\exp {\left( -\frac{(s\phi )}{\mu }y\right) }$	$\frac{\Lambda }{1-\frac{1}{\Gamma (s\phi )}\gamma (s\phi ,(s\phi /\mu )y)}$	$\eta =\log (\mu )$	$s,\phi >0$
Weibull	$\alpha y^{\alpha -1}\lambda \exp {(-\lambda y^{\alpha })}$	$\alpha y^{\alpha -1}\lambda$	$\eta =\log (\lambda )$	$\alpha ,\lambda >0$
Log-normal	$\frac{1}{y\sqrt{(2\pi )}}\sqrt{\tau }\exp {\Big (-\frac{1}{2}\tau (\log y -\mu )^2\Big )}$	$\frac{\frac{\sqrt{\tau }}{\sqrt{(2\pi )} }\exp {(-\frac{1}{2}\tau \left( \log y -\mu \right) ^2}}{1-\psi (\sqrt{\tau }({\log y-\mu )}{})}$	$\eta =\mu$	$\tau >0$
Cox	-	$h_0(y)\exp (\mu )$	$\eta = \mu$	-

In the Cox proportional hazard model [22], the hazard rate is defined as

5 $$\begin{aligned} h(y_{ij}) = h_0(y_{ij})\exp {(\eta _{ij})}, \end{aligned}$$

where $h_0(.)$ is the baseline hazard function. For a given partition of time *t* of death into *K*, we assume $h_0$ to be constant over each time interval. That is

6 $$\begin{aligned} h_0(t)=\exp (c_k), \,t\in (t_k-1,t_k],\,\,k=1,2,...,K. \end{aligned}$$

The parameters $c_k,k=1,2,...,K$ are unknown and estimated from the data. The prior model assigned to $c_k$ is discussed later in this section.

The performance of each model was evaluated using the Deviance Information Criterion (DIC) and the Watanabe Akaike Information Criterion (WAIC). The lower the criteria values, the better the model, [23]. The best parametric model among the competing models is used to quantify the impact of risk factors and spatial patterns on the hazard rate among under-age five children in Nigeria. Thus, we adopted a Bayesian hierarchical modeling framework.

Given the structural additive linear predictor,

7 $$\begin{aligned} \eta _{ij}=\mathbf{x}_{ij}^T\boldsymbol{\beta }+\mathbf{z}_{ij}^T\boldsymbol{\psi }+\mathbf{w}_{ij}^T\boldsymbol{\theta }, \end{aligned}$$

where $\boldsymbol{\beta }$ is a $p\times 1$ vector of linear effects, such as the mother’s education level, household wealth index, place of settlement, e.t.c. $\mathbf{x}$ is a $p\times 1$ design vector for the linear covariates. $\boldsymbol{\psi }$ is a $r\times 1$ vector of non-linear effects to account for variables that varies non-linearly with the time of death, such as the mother’s age. $\mathbf{z}$ is a $r\times 1$ design binary vector that links the corresponding effect parameters to the time of death. Finally, $\boldsymbol{\theta }$ is a $37 \times 1$ vector of spatial effects to account for heterogeneity between states in Nigeria, and $\mathbf {w}$ is a $37\times 1$ vector of covariates to incorporate spatial intercepts.

#### Prior models

In a Bayesian framework, the parameters in the linear predictor in Equation 7 are considered random, that is, a probability distribution is used to represent the uncertainty in the parameters. Hence, a non-informative multivariate normal distribution was assigned for $\varvec{\beta }$, that is, $\varvec{\beta }\sim N_p(\mathbf{0}, 100\mathbf{I})$, where $\mathbf{I}$ is a $p\times p$ identity matrix. Since the mothers’ age is equally spaced, a random walk of order two prior distribution was assigned for the non-linear effect, $\boldsymbol{\psi }$. Let $\boldsymbol{\psi }=(\psi _1,\psi _2,...,\psi _r)^T$, defined

8 $$\begin{aligned} \Delta _v=\psi _v-2\psi _{v+1}+\psi _{v+2}\sim N(0,\tau _{\psi }^{-1}),\,\,v=1,2,...,r. \end{aligned}$$

Thus, the joint prior distribution, given by [24] is written as

9 $$\begin{aligned} \pi (\boldsymbol{\psi }|\tau _\psi )\propto (\tau _\psi )^{(n-2)/2}\exp \Bigg \{-\frac{\tau _{\psi }}{2}\sum\limits_{v=1}^{r-2}(\Delta _{v})^2\Bigg \}\cdot \end{aligned}$$

The set of mothers’ ages, *R*, was ordered at regular intervals, that is, $R=\{ 15, 16,..., 49\}$. To facilitate estimation, R was re-coded into $R=\{ 1, 2,..., 35\}$, and $r=35$. Due to rank deficiency, a sum-to-zero constraint is employed. The random walk two is flexible, invariant under the addition of linear term to $\boldsymbol{\psi }$, and computationally flexible as it exhibits Markovian property [24].

To account for the spatial effects, the Intrinsic Conditional Autoregressive prior distribution was adopted. Let $\boldsymbol{\theta }=(\theta _1,\theta _2,...,\theta _{37})$, that is, $\boldsymbol{\theta }$ accounts for the spatial intercept and heterogeneity between the states. Let $\boldsymbol{\theta }$ be represented by a Gaussian Markov random field (GMRF) with respect to the Graph $\mathcal G=(\mathcal V,\mathcal E)$, where $\mathcal V$ is a set of vertices representing the 37 states and $\mathcal E$ is the set of edges connecting the states, thus, for $i\ne i^*$, $\{i,i^*\}\in \mathcal E$ if and only if state *i* and $i^*$ are neighbors. Let *W* be a $37\times 37$ adjacency matrix such that $w_{ii^*}=1$ if $\{i,i^{\star }\}\in \mathcal E$ and zero otherwise. Let $\mathbf{D}$ be a diagonal matrix, where each diagonal entry $d_{ii}$ equals the total numbers of neighbors of state $i\in \mathcal V$. Therefore, the precision matrix for the GMRF is given as

10 $$\begin{aligned} Q=\tau _{\theta } \mathbf{D}(\mathbf{I}-\mathbf{W}). \end{aligned}$$

Notice that by construction, *Q* is sparse, and hence, $\boldsymbol{\theta }$ is a Gaussian Markov random field with respect to $\mathcal G$. That is, $\theta _{i}$ and $\theta _{i^*}$ are conditionally independent given $\boldsymbol{\theta }_{-ii^*}$ if and only if $\{ i,i^* \}\notin \mathcal E$. Therefore, the prior probability density function is given as

11 $$\begin{aligned} \pi (\boldsymbol{\theta }|\tau _\theta )\propto \log (|Q|)\Big (-\frac{1}{2}\boldsymbol{\theta }^T Q\boldsymbol{\theta }\Big ). \end{aligned}$$

For the baseline hazard function Eq. (6), a first-order random walk was assigned for $c_k,\, k=1,2,...,K$. The definition is analogous to Eq. (8). Hence,

12 $$\begin{aligned} \Delta _k= & {} c_k-c_{k-1}\sim N(0,\tau ^{-1}_{c}),\nonumber \\ \pi (\boldsymbol{\mathbf c }|\tau _{c})\propto & {} (\tau _c )^{(n-1)/2}\exp \Bigg \{-\frac{\tau _{c}}{2}\sum\limits_{k=1}^{r-1}(\Delta _{k})^2\Bigg \},\\\mathbf c=&(c_1,c_2,...,c_K)^T. \end{aligned}$$

A log gamma hyperprior was assigned to $\log \tau , \log \tau _c,\log \tau _\psi ,\log \tau _\theta$, with hyperparameters $(1,5e^{-5})$. A $\log$ gamma prior model was assigned to $\log \phi$ with parameter (1, 0.01), and $\alpha$ was assigned a PC-prior distribution given as

13 $$\begin{aligned} \pi (\alpha )=\frac{\lambda }{2}\exp {(-\lambda d(\alpha ))}\left| \frac{\partial d(\alpha )}{\partial \alpha }\right| , \end{aligned}$$

where $d(\alpha )=\sqrt{2 KLD (\alpha )}$, and $KLD(\alpha )=(\Gamma ((1+\alpha )/2)\alpha +\alpha \log (\alpha )-\alpha \gamma +\gamma -\alpha )/\alpha$, $\gamma$ is the Euler’s constant, and $\lambda =5$.

#### Bayesian estimation

To estimate the parameters of interest, the structural additive linear predictor, (Equation 7), is used to formulate a latent variable $\mathcal X=\{\boldsymbol{\beta },\boldsymbol{\psi },\boldsymbol{\theta }\}$ having a multivariate Gaussian distribution with a sparse precision matrix $\mathbf{Q}(\boldsymbol{\phi }_1)$. Let $\boldsymbol{\phi }_2$ be a vector of all the hyperparameters of the likelihood function and the prior distributions, and $\boldsymbol{\phi }=(\phi _1,\phi _2)$ with joint distribution $\pi (\boldsymbol{\phi })$. Thus, the joint posterior distribution is given as

14 $$\begin{aligned} \pi (\mathcal X,\boldsymbol{\phi }|\mathbf{y})\propto \pi (\boldsymbol{\phi })\pi (\mathcal X|\boldsymbol{\phi })L(Y\mid \mathcal {X},\boldsymbol{\phi }), \end{aligned}$$

where $L(Y\mid \mathcal {X},\boldsymbol{\phi })$ is the adequate likelihood function given in Equation 4. The interest is to make inferences from the posterior marginal distributions $\pi (\mathcal X|\mathbf{y})$ and $\pi (\boldsymbol{\phi }|\mathbf{y})$. The formulation (Equation 14) can be estimated using Integrated Nested Laplace Approximation (INLA) described in [25, 26]. The parameters of interest were estimated using the R-INLA package in R [27]. The R-INLA package has been used as an estimation tool in several analyses across different study fields and has been shown to perform adequately, [28–30]. R-INLA estimates the posterior marginal distribution of the parameters of interest for inference. The estimation procedure is fully discussed in [23, 29, 31–33]. For the non-linear and spatial effect estimates, the output of the R-INLA provides the posterior marginal distribution for each non-linear variable and each administrative state, which is then summarized and graphically displayed using adequate functions in R. Given that a sum to zero constraints was imposed on the spatial effect, the probability of excess risk was computed from the posterior marginal distribution of the spatial effect, which is given as

15 $$\begin{aligned} \pi (\theta _i < 0 | \mathbf{y}) = \int _{-\infty }^{0}\pi (\theta _i |\mathbf{y})d \theta _i,\,\,i=1,2,...,37, \end{aligned}$$

where $\pi (\theta _i|\mathbf{y})$ is the posterior marginal distribution of the *i*th administrative state spatial effect. The interpretation of $\pi (\theta _i|\mathbf{y})$ varies between the models and the link functions adopted in the model specification. For the log-normal and gamma model, a higher probability, $\pi (\theta _i|\mathbf{y})$, indicates a lower risk of death or a higher probability of a child residing in state *i* to survive past age five. Whereas, for Cox proportional hazard, exponential, and Weibull model, a lower value of $\pi (\theta _i|\mathbf{y})$ indicates a lower risk of death or a higher probability a child residing in the state *i* to survive past age five. Moreover, the integral in Equation 15 was approximated using Monte Carlo samples from the marginal posterior distribution. That is

16 $$\begin{aligned} \pi (\theta _i<0 | \mathbf{y}) \approx \frac{\sum\nolimits_{l=1}^{N}I(\theta _{il}< 0)}{N}, \end{aligned}$$

where $\theta _{il}$ is the *l*th sample from $\pi (\theta _i |\mathbf{y})$, *I*(.) is an indicator function and $N=10,000$.

## Results

Among the 33, 697 children included in the analysis, the estimated censoring rate is $90\%$. It indicates that for every 1000 live birth, 100 children never attained their fifth birthday. The descriptive statistics of the data used are shown in the Appendix.

### Nonparametric method

**Fig. 2:**
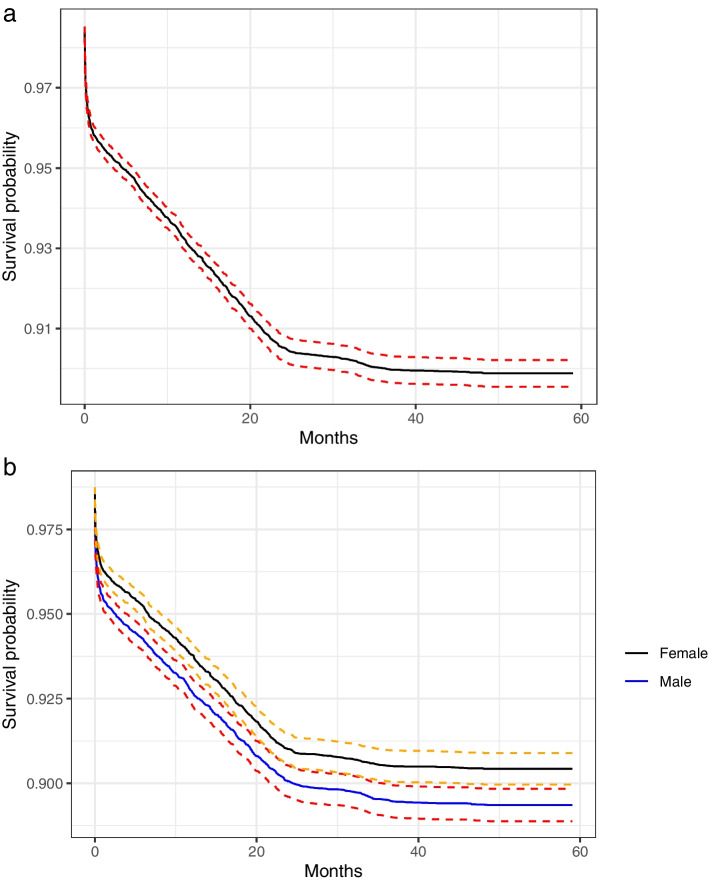
**a** Kaplan-Meier survival probability estimate, **b** Kaplan-Meier survival probability estimate accounting for gender

**Fig. 3:**
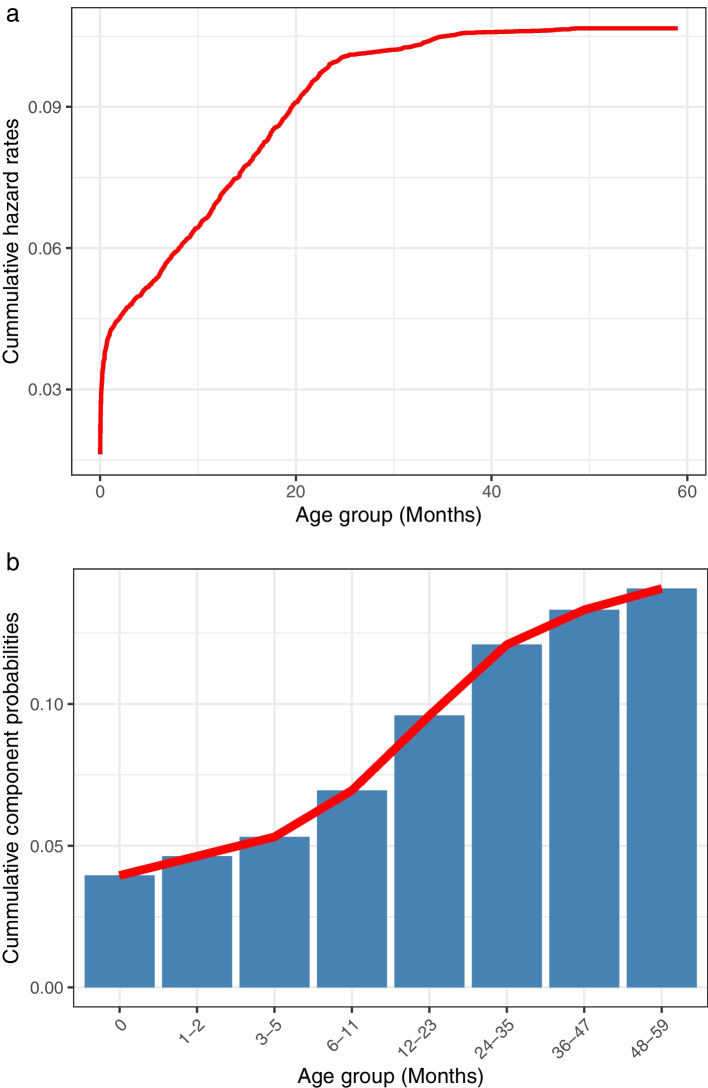
**a** Nelson Aalen cumulative hazard curve, and **b** cumulative component mortality probability estimate. The highest hazard rate and highest component mortality probability are within the first two months after birth

Figure 2a presents the overall Kaplan-Meier survival probability estimates without taking any covariate effects into account. The middle black line indicates the mean survival probability, while the red lines indicate the 95% confidence interval of the survival probability. The curve shows a sharp drop in the survival probabilities among children within the first two months, and further consistently drops at a constant rate until the age of 25th months. It slowly descends further until a child attains 40 months, and then levels off at a probability of 0.88. The probability of a child surviving past the first two months declines significantly, and consequently, the probability of dying within this period increases with a magnitude of at least $1-0.96=0.04$. In Figure 2b, the gender effect was taken into account. Though the survival curves for both males and females relatively have a similar pattern, the survival probability of male children is lower compared with that of female children. While the survival curve for females levels off at 0.906, the curve for male children leveled off at 0.894 before the fifth birthday. This implies that male children are more likely to die, with a probability of $1-0.894=0.106$ compared with female children with a probability $1-0.906=0.094$. Figure 3a presents the cumulative hazard rate computed using the Nelson Aalen estimator of cumulative hazard curve, and Figure 3b presents the cumulative component probabilities of a child dying before reaching 59 months at each age group. In Figure 3a, there is a sharp increase in the cumulative hazard rates within the first two months after birth. The curve then increased at a constant pace until 25 months and then gradually levels off until 59 months. In Figure 3b, the highest component probability occurred within the first months after birth. It is followed by the probability associated with a child crossing from the age group $3-5$ to $12-23$ months and $12-23$ to $24-26$ months.

### Parametric method

Table 2 shows the DIC and WAIC information criteria. Based on these measures, the log-normal model is the most appropriate model for the analysis, and thus, only the results of the log-normal model are presented. However, the presented result is consistent for all the fitted models. It is important to mention that the interpretation of the models’ effects varies between the models considered and depends on the link function adopted (see Table 1). For the log-normal model and the link function considered in this study, an increased value of model effects implies decreased hazard rate and increased survival probability. Thus, factors with lower effects (coefficients) are considered risk factors for under-five mortality.

**Table 2:** Model adequacy. The Log-normal model has the least DIC and WAIC, and thus, it is the best model among the competing models

Model	DIC	WAIC
Exponential	31477.63	22858.4
Gamma	17102.27	17003.58
Weibull	26064.64	16683.35
Log-normal	15784.98	15679.97
Cox	126512.6	9.277956e+62

#### Linear effect

The linear effect estimates are presented in Table 3, showing the posterior mean, standard deviation (sd), the lower (2.5%), and upper (97.5%) quantile of the 95% credible interval. The reference category for the categorical variables is indicated as “Reference”. The non-inclusion of zero in the credible interval was used to determine significance.

**Table 3:** Posterior estimates of the fixed effects

Variables	Posterior mean	Posterior sd	2.5% CI	97.5% CI
Gender				
Male	Reference			
Female	0.226	0.035	0.157	0.294
Maternal Education				
No formal education	Reference			
Primary	0.153	0.056	0.043	0.263
Secondary	0.209	0.054	0.103	0.315
Higher	0.253	0.091	0.074	0.432
Wealth quantile				
Poorest	Reference			
Poorer	0.106	0.049	0.009	0.203
Middle	0.177	0.056	0.067	0.287
Rich	0.277	0.066	0.147	0.407
Richest	0.409	0.082	0.249	0.569
Source of water				
Unimproved	Reference			
Improve	0.078	0.037	0.006	0.151
Toilet facility				
Unimproved	Reference			
Improve	0.100	0.042	0.017	0.182
Residence				
Urban	Reference			
Rural	0.169	0.041	0.089	0.250
Mass media usage				
No	Reference			
Yes	0.077	0.039	0.000	0.155
Frequency of Antenatal care visit				
No visit	0.474	0.064	0.348	0.600
2-4	Reference			
$5+$	0.071	0.044	-0.015	0.156
Frequent postnatal care visit				
No	Reference			
Yes	0.246	0.065	0.118	0.374
Child was being breastfed				
Yes	Reference			
No	-3.207	0.064	-3.332	-3.082
Place of delivery				
Health facility	Reference			
Home	0.220	0.042	0.137	0.303
Mother has being working in the last 12 months				
No	Reference			
Yes	0.181	0.038	0.106	0.255
Marital status				
Never Married	Reference			
Married	0.394	0.053	0.290	0.499
Divorced/Spouse Diseased/Separated	0.388	0.116	0.160	0.617
Religion				
Other Christian	Reference			
Catholic	0.264	0.079	0.109	0.420
Islam	0.139	0.049	0.043	0.234
Traditional	0.156	0.312	-0.444	0.778
Others	0.414	0.374	-0.303	1.166
Birth				
Single birth	Reference			
Multiple birth	-0.875	0.077	-1.025	-0.724
Birth orders				
First birth	0.103	0.055	-0.006	0.212
2-4	Reference			
5+	-0.046	0.047	-0.139	0.047
Mode of delivery				
Normal	Reference			
Cesarean	0.040	0.116	-0.187	0.269
Tetanus Injection				
No	Reference			
Yes	0.531	0.043	0.447	0.615
Household have bednet				
No	Reference			
Yes	-0.178	0.044	-0.264	-0.093
Children sleeps under bednet				
No	Reference			
Yes	0.529	0.042	0.446	0.611
Who decide health care use				
Respondent alone	Reference			
Both	0.160	0.056	0.051	0.269
Spouse	0.230	0.050	0.132	0.327
Someone else	-0.226	0.341	-0.887	0.451
Access to health care				
Limited Access	Reference			
Full Access	-0.038	0.292	-0.603	0.543

The result revealed that female children are significantly less likely to die before their fifth birthday compared with male children, which is the counterpart category. This result is consistent with the Kaplan-Meier estimates. Children with mothers who achieved at least a primary level of education are more likely to attain age five compared with children whose mothers did not have any formal education. Moreover, as mothers’ education increases, the more likely a child will celebrate the fifth birthday. A similar pattern is found for the household wealth quantile. Children whose household wealth quantile is at least poorer are significantly more likely to attain the fifth birthday compared with the poorest category. As the wealth quantile increases, the more likely a child would survive the fifth birthday.

Children living in a household with improved water and toilet facility are significantly more likely to celebrate their fifth birthday compared with their counterpart, which is unimproved water and toilet facility. The result shows that children living in rural areas are significantly more likely to attain the fifth birthday compared with their counterparts living in urban settlements. Children whose parents pay attention to at least one mass media means are significantly more likely to attain age five. Children whose parents visit antenatal care more than five times before birth is not significantly different from those whose parents had 2-4 visits before childbirth. Surprisingly, children whose parents do not visit antenatal care are more likely to attain age five compared with the base category. However, for postnatal care utilization, this is not the case. Children whose parent frequently visits postnatal care are significantly more likely to attain age five.

Children not being breastfed are significantly more likely to die before their fifth birthday. Unexpectedly, the result indicated that home-delivered children are less likely to die before age five compared with their counterparts who were delivered in a health facility. Children whose mothers have been working (employed) in the last twelve months are more likely to attain age five compared with children whose mothers have not been working in the last twelve months before the survey. For the type of religion, children whose religion is Catholic, Islam, Traditional, and Others are more likely to attain their fifth birthday compared with the base category, which is other Christians. Children whose birth was multiples are more likely to die before age five. However, the birth position is not significant.

The result indicates that the mode of delivery does not significantly contribute to the survival of a child. As expected, children who took tetanus injections are less likely to die before age five compared with children who do not. Surprisingly, children whose household has a bednet are more likely to die before the fifth birthday compared with children whose household does not have a bednet. However, children who sleep under bednets are less likely to die before age five. Children whose both parents or spouses decide the health care use are more likely to attain the fifth birthday compared with the children whose health care use is only decided by the respondent alone. Moreover, children whose health care use is decided by someone else are not significantly different from the base category. Access to a healthcare facility does not significantly improve the likelihood that a child would survive age five.

#### Spatial effect

**Fig. 4:**
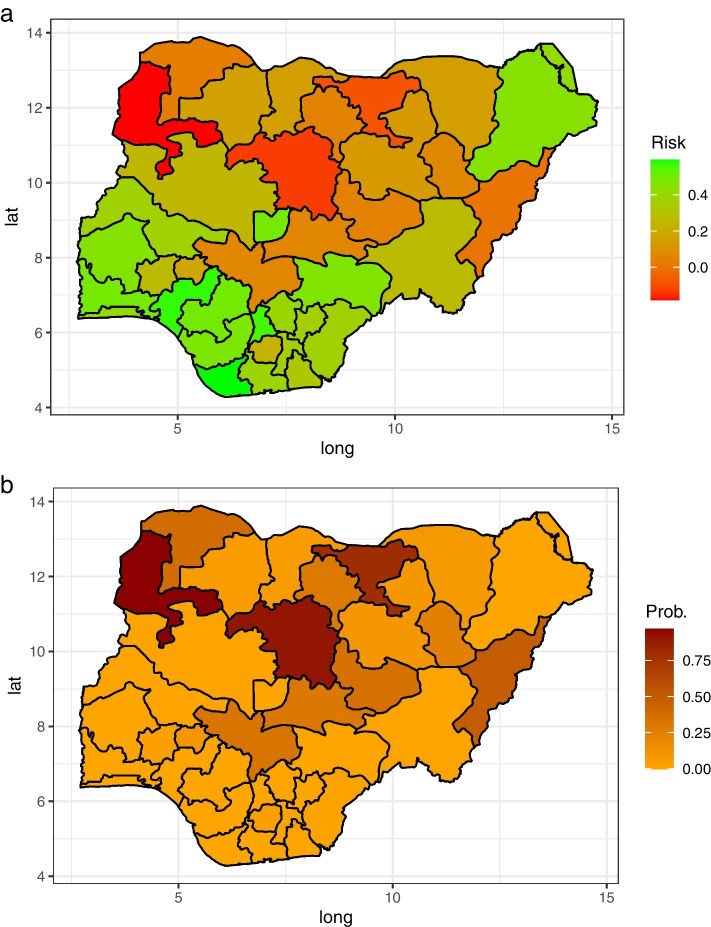
**a** The spatial effect of state base survival risk, and **b** is the probability of elevated risk of mortality before attaining the fifth birthday. The red states indicate regions with a higher mortality risk than the overall average. States with higher probability indicate regions with a higher probability of under-five death before attaining the fifth birthday

Figure 4 presents the posterior estimate of the spatial effect of a child dying before the fifth birthday. In Figure 4a the reddish States represent the states with a higher likelihood of under-five mortality, while the greenish States are regions with a lower likelihood relative to the overall country’s average. Figure 4b presents the probability of elevated risk of a child dying before attaining age five. In general, the northern region of Nigeria is at a higher risk of under-five mortality. In other words, the likelihood of child mortality is more severe in the North. Among the Northern states, Kebbi, Kaduna, and Jigawa states have above 0.70 probability of elevated risk of under-five mortality compared with the overall average risk. That is, the likelihood of child mortality in these states is above $70\%$ higher compared with the country’s average. Invariably, Kebbi, Kaduna, and Jigawa can be tagged as child mortality hotspots in Nigeria. These are followed by Adamawa, Gombe, Kano, Kogi, Nasarawa, Plateau, and Sokoto states. However, turning attention to the southern region of Nigeria, Ekiti, Imo, and Osun states have below 0.25 probability of having an elevated risk of a child dying before attaining age five compared with the overall average risk. It indicates that the likelihood of under-five mortality in these states compared with the country’s average is below $25\%$.

#### Non-linear effect

**Fig. 5:**
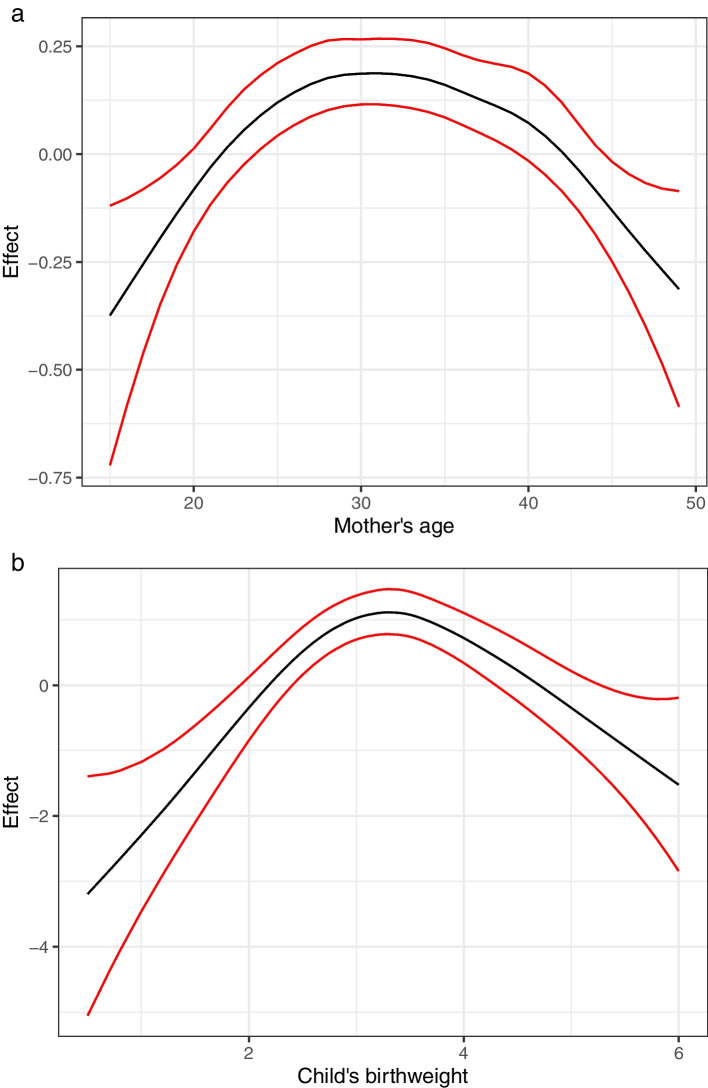
**a** The effect of maternal age (years), and **b** child’s birth weight (Kg) to under-five mortality before attaining the fifth birthday. The risk is higher at the beginning and end of maternal age, and birth weight below 2.5kg and above 4.5kg

Figure 5 presents the non-linear effects of maternal age and a child’s birth weight on child mortality before attaining the fifth birthday. In general, the shape of the effect of maternal age and birth weight on child mortality is an “n” shape. In Figure 5a the likelihood that a child will attain the fifth birthday increases gradually as the maternal age increases from 15 years and attains a peak at the age of 27 years, and maintains the peak for further 6 years, and then gradually decreases through age 35 to age 49 years. This indicates that there is a higher likelihood of child mortality among young women and older women compared with women within the age range of 24 to 44 years. Turning attention to Figure 5b, the likelihood of a child to attain the fifth birthday gradually increases from a birth weight of 0.5 kg until it attains a peak at 3kg and maintains the same effect for an additional 0.5kg. The likelihood gradually decreases until a birth weight of 6kg. In other words, the likelihood of child mortality is higher among birth weights less than 2.5kg and those higher than 4.5kg.

## Discussion

This study considered the time-to-death outcomes of under-five children in Nigeria using both parametric and nonparametric methods. The results of the adopted nonparametric methods were consistent, and it indicates a higher risk of under-five mortality within the first two months after birth and thus, should be considered as a hot period in time localization. Improving the well-being of children at this stage could significantly scale down the child mortality index in Nigeria. Moreover, among all the parametric models considered, the log-normal model stood out as the most suitable model to explain the variations in the time-to-death outcomes variable. Findings show that gender, maternal education, household wealth status, source of water and toilet facility, residence, mass media, frequency of antenatal and postnatal visits, marital status, place of delivery, multiple births, who decide healthcare use, use of bednet are significant risk factors of child mortality. Findings also indicate a disparity in geographical regions, across mothers’ ages, and birth weights.

Findings show that the survival probability of under-five children has the highest drop within the first two months after birth. That is, children within the first two months of birth have the highest expected risk of death. This finding corroborates the result conducted in rural Burkina Faso, [34], and Ethiopia, [35]. Hence, this study suggests that intervention programs set to mitigate mortality should give priority to children within the first two months as this could effectively scale down the overall under-five mortality index in Nigeria. Findings indicate that male children have an elevated risk of dying before their fifth birthday compared with female children in the same age group. This finding is consistent with biological results that male children are biologically weaker and more susceptible to diseases compared with female children, [36, 37]. Also, it may suggest that male children are more vulnerable to health inequalities which is more noticeable in the early stages after birth, [38]. Findings showed that attainment of higher maternal education increases the survival chances of children. This finding is expected as educated parents are more likely to have a positive attitude towards parenting, often seek expert advice, and involve in a child’s physical and mental growth, [39, 40]. Thus, this finding suggests that maternal education is among the key important markers to mitigating the high mortality rate in Nigeria.

This study identified a discrepancy in the risk of mortality among households’ wealth status. That is, the risk of mortality is most severe among the poorest wealth quantile, and the risk consistently decreases with an increase in wealth quantile. The most prominent increase in under-five survival was found for a one-step wealth quantile increase from the rich to the richest. However, for intervention, improving household wealth quantile from rich to richest seems impractical, rather programs could focus on the improvement from poorest to poorer quantile by providing basic living conditions, such as access to good shelter, roads, and electricity being that the improvement from poorest to poorer wealth quantile is the second most prominent increase in under-five survival probability. This finding is consistent with existing studies, [41, 42]. Wealth inequalities, non-diversification of the economy, unemployment, and low education financing in Nigeria are the major key factors contributing to increasing poverty, [43], and consequently, increase the risk of child mortality in Nigeria. This finding identifies poverty as a leading cause of mortality. Poverty alleviation programs to improve the livelihoods of households are one of the main remedies to child mortality in Nigeria.

Findings also indicated that access to an improved water source and toilet facility lowers the risk of mortality. However, the improvement is more prominent for the toilet facilities. Thus, policies to safely managed improved household water sources and toilet facilities are required to mitigate child mortality, [44]. Surprisingly, the frequency of antenatal care visits does not improve the survival probabilities of children. However, the survival probability increases for postnatal visits. It could be associated with late first-time antenatal utilization or irregular visitation. Women tend to wait towards the delivery period or complications during gestation before utilizing antenatal care services. Thus, educating mothers on the importance of early antenatal utilization could scale down the under-five mortality rate. Similarly, home delivery in Nigeria significantly improves the survival probabilities of a child to celebrate the fifth birthday. This finding is contrary to the common rationale that babies delivered in a health facility are more likely to attain their fifth birthday since medical experts are readily available to give the first medical support and advice. According to [8], the prevalence of non-utilization of health facilities for childbirth is about 62% of the total birth in Nigeria. It could be associated with poor quality of health facility, caring, cost, and cultural beliefs that lack the acceptability of health care services, and the belief that healthcare facility is only necessary during birth complications, [45]. Thus, intervention programs that give financial support and prenatal education are needed to encourage the frequent use of health facilities but not only when there are complications during childbirth.

Findings indicate that maternal occupation since a year before the survey significantly lowers the risk of child mortality. This finding corroborates the previous study, [46]. However, the reverse may be the case if only children under breastfeeding were considered, [47]. Findings indicate that married couples increase the survival probability of children attaining their fifth birthday. Nonetheless, divorced, deceased spouses or separated parents also increase survival compared to never-married parents. It could be linked to the elevated likelihood of children of unmarried parents suffering the consequence of limited resources, poverty, and poor schooling, [48, 49]. This study also found that multiple births significantly increase the risk of under-five mortality compared with a singleton. It could be associated with the consequence of shared limited family resources among the children. This finding suggests that multiple births jeopardize the survival of under-five children. Hence, children of multiple births should be given special treatment in addition to positive involvement in parental support. Findings also indicate that immunization with Tetanus strongly increases the survival of children. This finding is in line with the result of a previous study, [50]. Thus, immunization with Tetanus and its benefits on the under-five survival should be well publicized and target parents with low maternal education attainment. This study found that household with bednets does not lower the risk of child mortality. However, the usage of insecticide-treated bednets among children significantly lowers the risk of mortality in children. The magnitude of the effect of bednet use in the improvement of child survival is equivalent to the magnitude of immunization with Tetanus. This finding suggests that the non-usage of insecticide-treated bednet increases the risk of mortality, and it should be considered as a significant risk factor. Thus, intervention programs that donate and distribute insecticide-treated bednets to households in Nigeria should take it as an additional duty to educate parents on the importance of the use of bednets on the survival of children. This finding is consistent with a previous study in Ghana, [51]. This study found that both parent and husband’s decision on the mother’s health use significantly improves child survival compared to mother decision alone. Moreover, a someone-else decision on the mother’s use of health services increases the risk of mortality. This finding suggests that decisions on mother’s health use outside the parents could increase the risk of mortality. Hence, at least spouses (fathers) should be involved in the decision-making on health use. The role of the father to a child’s health plays a crucial part in child survival, as studies have shown that the father’s involvement is related to positive child health outcomes, [52].

This study also substantiates the effect of maternal age and birth weight on child mortality. Findings indicate that the risk of mortality among under-age five children increases for maternal age below 24 and above 44 years. This finding suggests that maternity age in the mentioned disjoint age intervals is a risk factor for child mortality. For younger women, this could be attributed to a lack of experience with a child’s nutritional needs or could be attributed to the biological and social mechanism, [53]. Whereas for older women, it could be attributed to the prevalence of complications during childbirth and preterm delivery, [54]. Similarly, the likelihood of under-age five mortality is high at the lowest birth weights, improves within the most frequent average birth weights, and then picks again at extremely higher birth weights. Specifically, it was found that birth weight less than 2.5kg and above 4.5kg elevates the risk of underage five mortality. It could be attributed to the prevalence of subnormal growth and neurodevelopmental complications among children with low birth weight, [55]. These findings are consistent with previous studies, [56, 57].

This study identified significant spatial heterogeneity in the risk of under-five mortality before attaining the fifth birthday. Findings show a clear divide between North and South Nigeria, placing the Northern states at higher risk regions compared with the South. Specifically, among the Northern states in Nigeria, this study identified Kebbi, Kaduna, and Jigawa as the hotspot of child mortality in Nigeria. Additionally, Adamawa, Gombe, Kano, Kogi, Nasarawa, Plateau, and Sokoto states were also identified as high-risk regions. This finding corroborates the work of [15].

This study has some limitations. Firstly, regional factors that directly influence the survival probabilities of children were not available in the data. These factors include variables related to food security and child nutritional intake, which can play key roles in a child’s survival. Moreover, there is a nonavailability of data on the cause of child death to account for the risk of unavoidable deaths and its impact on the research results. Secondly, the adopted model could suffer from the omission of diseased children from the survey and the missed information on the exact day of death, which consequently may affect the point survival probability estimates. Lastly, the multi-stage design of the survey was not incorporated in the estimation stage. Future work can consider such implementation to verify whether there is a sufficient improvement in the results relative to the complexity it introduces into the model.

## Conclusion

This study quantified the risk of under-five mortality in the form of survival probabilities before a child attains the fifth birthday, and also identified the risk factors elevating the under-five mortality rate in Nigeria. These findings call for action on the identified risk factors to mitigate the under-five mortality rate in Nigeria. This study accentuates the need for special attention for the first two months after childbirth as it is the age group with the highest expected mortality. A practicable way to minimize death in the early life of children is to improve maternal healthcare service, promote maternal education, encourage delivery in healthcare facilities, positive parental attitude to support multiple births, poverty alleviation programs for the less privileged, and a prioritized intervention to Northern Nigeria. The identified hotspot could be used for policy-making and as a guide towards the distribution of intervention resources. Additionally, it could serve for the construction of a localized framework for intervention program evaluation. The maps produced in this study could be placed beside another map, such as poverty, unemployment rate, or insurgence maps to identify possible correlation across the country.

## Data Availability

The Nigeria Demographic and health survey data used in this study are available and accessible on the DHS program website (http://dhsprogram.com).

## Abbreviations

DIC: Deviance Information criterion
EA: Enumeration Areas
NDHS: Nigeria Demographic and Health Survey
NPC: National Population Commission
R-INLA: R-Integrated Nested Laplace Approximation
UNICEF: United Nations Children’s Fund
WAIC: Watanabe-Akaike Information Criterion
WHO: World Health Organization
